# Endoscopic Ultrasound-assisted Diagnosis of Obscure Gastrointestinal Bleeding

**DOI:** 10.7759/cureus.5581

**Published:** 2019-09-06

**Authors:** Kapil Gupta, Issam M. Kably, Mohit Girotra

**Affiliations:** 1 Internal Medicine, University of Miami/JFK Medical Center, Atlantis, USA; 2 Interventional Radiology, University of Miami, Miami, USA; 3 Gastroenterology and Hepatology, University of Miami, Miller School of Medicine, Miami, USA

**Keywords:** hemosuccus pancreaticus, gastrointestinal bleed

## Abstract

Hemosuccus pancreaticus (HP) describes hemorrhage originating from the pancreatic duct. HP is an extremely uncommon source of upper gastrointestinal bleeding and is often misdiagnosed in most community hospitals. HP is believed to be associated with arterial aneurysm, pancreatitis (acute or chronic), local inflammation, pseudocyst, and tumor or cystic neoplasms. We report a case of a 62-year-old man with multiple cysts in the pancreas on CT scan who presented with an obscure upper GI bleed in which we performed step-wise investigations that led us to the diagnosis of hemosuccus pancreaticus, which was made through endoscopic ultrasound. After diagnosis, the patient was treated successfully by interventional radiology.

## Introduction

Hemosuccus pancreaticus (HP), also called wirsumgorrhagia, alludes to hemorrhage originating from the pancreatic duct [1]. HP is an extremely uncommon source of upper gastrointestinal bleeding (GIB), accounting for about 1/1500 cases of all GIB, and is often misdiagnosed in most community hospitals [2]. The clinical presentation of HP may include anemia, recurrent GIB (melena) and/or abdominal pain in the setting of normal pancreatic and liver enzymes [3]. HP may be related to pancreatic trauma, neoplasm, infection or vascular etiology like aneurysm, pseudoaneurysm, vasculitis, or lastly iatrogenic. If undiagnosed, HP can be potentially life-threatening, and hence needs astute clinical suspicion [3].

To elicit the challenges in diagnosis, and various modalities available for management, we discuss an interesting case, where HP was undiagnosed for several months, and ultimately endoscopic ultrasound (EUS) was helpful in securing an accurate diagnosis.

## Case presentation

**Figure 1:**
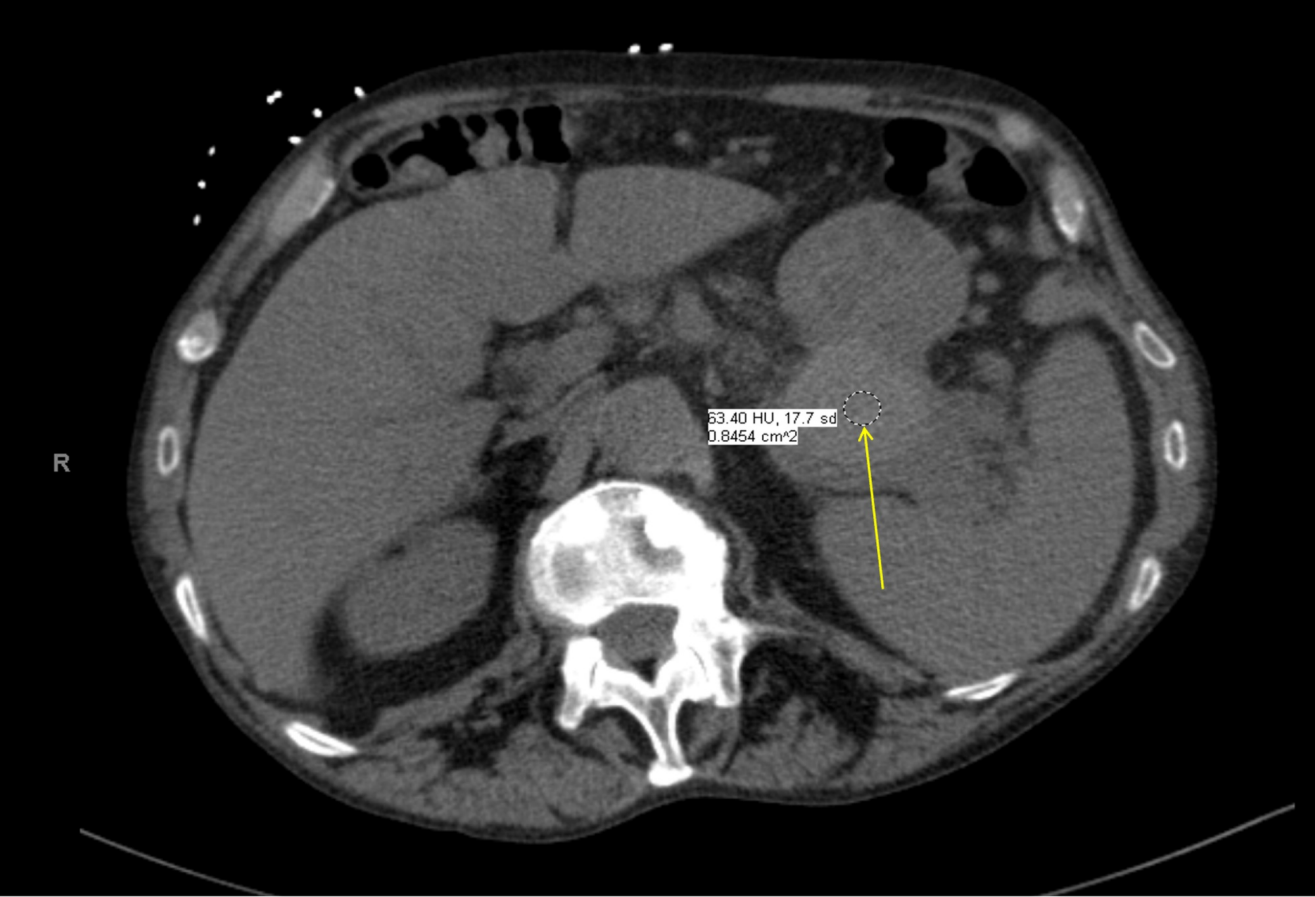
CT scan of the abdomen with peri-pancreatic stranding and multiple small fluid collections The arrow points to the largest collection in the superior aspect of the pancreatic tail measuring 4.7 x 3.4 cm.

A 62-year-old man with a past medical history significant for iron deficiency anemia, coronary artery disease, benign essential hypertension, gastroesophageal reflux disease, treated hepatitis C, alcoholic liver cirrhosis with history of esophageal varices, and internal hemorrhoids was a well-known patient for our hepatology team for history of liver cirrhosis in the past. Four months prior to establishing care in our clinic, he had presented with epigastric pain to the emergency department. During that emergency department visit, he underwent a CT of the abdomen and pelvis with intravenous contrast, which was notable for peri-pancreatic stranding and multiple small fluid collections around the entire pancreas, the largest of which was along the superior aspect of the pancreatic tail measuring 4.7 x 3.4 cm (Figure 1).

**Figure 2:**
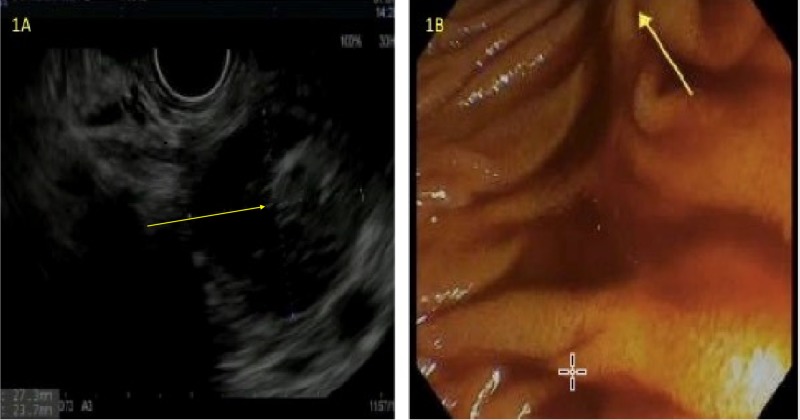
Endoscopic images (A. EUS, B. Upper esophagogastroduodenoscopy) (A) Endoscopic ultrasound (EUS) demonstrating complex pancreatic collection containing hyperchogenic blood-like material. (B) Endoscopic confirmation of bleeding from the ampulla.

Evaluation done at an other center showed a hemoglobin level of 5.9 and was admitted to the emergency department for workup. The patient gave a history of having intermittent melena and one episode of emesis of blood clot. The patient had a full evaluation with esophagogastroduodenoscopy (EGD) and colonoscopy to rule out common causes or expected causes of GI bleed. EGD showed small isolated gastric varices (IGV 1 in the fundus) without bleeding, and otherwise normal stomach mucosa and no esophageal varices. On colonoscopy, the patient had medium-sized non-bleeding external and internal hemorrhoids but the entire colonic mucosa was unremarkable. The terminal ileum was intubated and showed old coffee-ground appearing contents. His video capsule endoscopic examination was unremarkable. The patient was conservatively managed but the exact source of bleeding was not identified, and his bleeding stopped. Two weeks later, the patient underwent endoscopic ultrasound (EUS) to evaluate for chronic pancreatitis, and also follow up of pancreatic tail cyst found on imaging four months prior. EUS demonstrated an overall atrophic appearance of pancreas with diffuse hypoechogenicity with lobularity and honeycombing, and shadowing calcifications, with areas of hyperechogenic stranding, most prominent in the distal body and tail of the pancreas. Additionally was seen a round peripancreatic complex collection measuring about 27.3 x 23.7 mm, with some anechoic areas along with hyperechogenic contents, which was suspected to be old blood (Figure 2A). The pancreatic duct (PD) appeared normal, without any disruption or stricture, and no obvious communication with the cyst was identified, and the common bile duct (CBD) was unremarkable. Given complex nature of the collection, fine needle aspiration (FNA) was deferred.

For further evaluation of the complex pancreatic collection containing hyperchogenic blood-like material, with the background presentation of recurrent melena and persistent anemia, a duodenoscope was used to evaluate the ampulla, and small amount of blood was seen extruding, raising differentials for HP or hemobilia (Figure 2B). The upper enteroscopic examination to proximal jejunum was otherwise unremarkable. CT angiography (CTA) of the abdomen did not show any mass in the liver or biliary system, but was notable for pancreatic pseudocyst with no evidence of active arterial extravasation, findings suggestive of chronic pancreatitis, thrombosis of distal splenic vein (associated perisplenic and upper abdominal collaterals).

**Figure 3:**
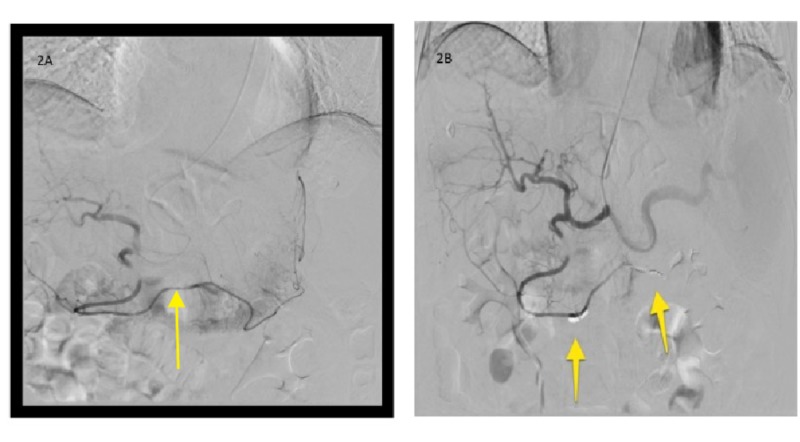
Angiogram images (A) Transradial selective catheterization and angiogram of the gastro-epiploic artery demonstrated an area of contrast blush and accumulation at the midsection of the artery without active extravasation, and distal communication with the splenic artery. (B) Post-embolization selective run-off shows superselective microcoils embolization using the “sandwich technique” distal and proximal (arrows) to the area of abnormal angiographic blushing resulting in immediate disappearance of the blush.

**Figure 4:**
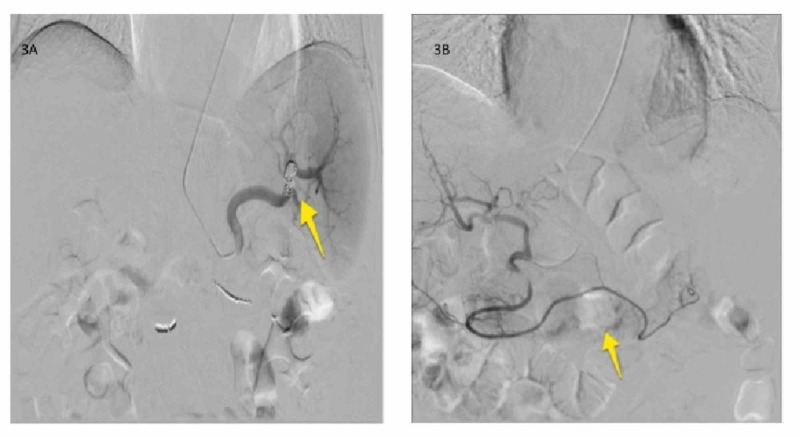
Embolization angiogram images (A) Transradial selective post-embolization angiogram of the splenic artery shows partial proximal embolization of the splenic artery with Penumbra™ POD detachable plug to further decrease the arterial pressure on the gastro-epiploic artery. The intra-parenchymal splenic vasculature remained patent. (B) Selective catheterization and angiogram of the gastro-epiploic artery demonstrated an area of contrast blush (arrow) and accumulation without active extravasation, and distal communication with the splenic artery.

After multi-disciplinary discussion, IR approach was preferred over endoscopic approach, and through ultrasound-guide access of the left radial artery, selective catheterization of the celiac trunk, with subselective catheterization of the left gastric artery/splenic artery/gastroepiploic artery branch of the gastroduodenal artery with angiogram was performed. Superselective gastroepiploic angiogram demonstrated abnormal area of contrast pooling at the level of the midportion of the gastroepiploic artery corresponding with site of pancreatic complex collection abutting the stomach (Figure 3A, 3B), and finally coil embolization of the gastroepiploic artery (bleeding source), and splenic artery (to decrease flow to the area of bleeding) was achieved with "back and front door" technique (Figure 4A, 4B). The patient did well post-procedure, and did not have further bleeding, and hemoglobin remained stable on two-month follow-up.

## Discussion

HP refers to bleeding originating from the ampulla of Vater through the pancreatic duct, but the bleeding source could be from the pancreas, pancreatic duct, or adjacent viscera/arteries [4]. Sandblom first described the entity in 1970 [1,5]. Patients with HP generally present with upper GIB symptoms: melena and/or hematemesis, and may additionally complain of having epigastric pain, which had been attributed to increased pressure in PD from blood clots [6]. The proposed pathophysiology of HP is the rupture of pseudoaneurysm by autodigestion of vessel wall by pancreatic enzymes or pancreatic cyst causing pressure necrosis leading to bleeding from splenic artery, gastroduodenal artery, and pancreaticoduodenal artery (in the order of prevalence) [2-4]. HP is believed to be associated with arterial aneurysm, pancreatitis (acute or chronic), local inflammation, pseudocyst, and tumor or cystic neoplasms [1,4]. Although in less frequency, cases have been reported in which pancreatolithiasis and/or pseudocysts have led to increased inflammation and ultimately HP. Consequently, given the relationship between pseudocysts and pseudoaneurysms, there is a belief that pancreatic enzymes play a pathophysiologic role as pancreatic pseudocysts contain activated lytic enzymes [4].

Diagnosis of HP is challenging, especially given the intermittent nature of bleeding. The workup must always first include an EGD to exclude more common causes of upper GI bleeding, and in cases of negative workup, side viewing duodenoscope must be utilized for detailed ampullary views [6]. If HP is suspected, duplex ultrasound may be helpful in initial evaluation of pancreatic pseudocyst and aneurysm/pseudoaneurysm. Contrast-enhanced computed tomography allows high-quality visualization of pancreatic architecture and concerning pathology (chronic pancreatitis, pseudocyst, pseudoaneurysm) [6]. However, the diagnostic standard remains angiography given its ability to discern the contributing artery, to determine its anatomy, and to act therapeutically [6].

The primary goal of management is the obliteration of the bleeding source, although some authors have proposed the idea of using pancreatic enzymes or drug therapy [4]. Mortality in untreated cases of HP may be as high as 90%. Interventional procedures have been effective as a first-line treatment in 67-100% of the cases, especially if angiography is able to locate the bleeding source. There are three methods of interventional radiologic procedures, including balloon tamponade, stent grafting, but coil embolization remains the most frequently discussed technique and can cause thrombosis of pseudoaneurysm [4]. For failed IR cases, surgical management can be considered [6], the choice of which depends on source/site of bleeding, and options include distal pancreatectomy, splenectomy, central pancreatectomy, intracystic ligation of the blood vessel, aneurysm ligation, and bypass graft [4,6]. Surgical options for hemorrhagic pseudocysts include excision of pseudoaneurysm and pseudocyst, and for technically challenging resections, ligation of the arteries proximal and distal to the pseudoaneurysm and/or pseudocyst can be undertaken. The downside of surgical management is high mortality of 20-25%, with overall success being 70-85% [6]. Endoscopic stent placement in the PD helps results in tamponade, and has been described in literature, but may be a temporary salvage technique, rather than a reliable solution [7]. EUS-guided angiotherapy is being discussed as a novel therapy for treating HP, as both a diagnostic and therapeutic alternative [8].

## Conclusions

Our case is a classic representation depicting both the challenges in diagnosing HP and the potential success of treatment through the IR approach. The novelty in our case was the high index of suspicion raised by thorough EUS examination that revealed hyperechogenic contents in the complex pancreatic cyst, raising concern for blood. The likely mechanism for HP in our patient was development of pseudoaneurysm secondary to an episode of pancreatitis followed by autodigestion of the vessel wall by pancreatic enzymes, and/or pressure necrosis by pseudocyst. The case also highlights the step-wise investigation algorithm needed for such obscure cases of UGIB, and stresses on the need for remaining cognizant for potential biliary and pancreatic sources of bleeding in such scenarios.
